# Design and Sensitivity Analysis Simulation of a Novel 3D Force Sensor Based on a Parallel Mechanism

**DOI:** 10.3390/s16122147

**Published:** 2016-12-16

**Authors:** Eileen Chih-Ying Yang

**Affiliations:** Advanced Institute of Manufacturing with High-tech Innovations, and Department of Mechanical Engineering, National Chung Cheng University, Chia-Yi 621, Taiwan; cyyang@ccu.edu.tw; Tel.: +866-5-272-0411 (ext. 33326)

**Keywords:** 3D force sensor, parallel mechanism, sensitivity analysis

## Abstract

Automated force measurement is one of the most important technologies in realizing intelligent automation systems. However, while many methods are available for micro-force sensing, measuring large three-dimensional (3D) forces and loads remains a significant challenge. Accordingly, the present study proposes a novel 3D force sensor based on a parallel mechanism. The transformation function and sensitivity index of the proposed sensor are analytically derived. The simulation results show that the sensor has a larger effective measuring capability than traditional force sensors. Moreover, the sensor has a greater measurement sensitivity for horizontal forces than for vertical forces over most of the measurable force region. In other words, compared to traditional force sensors, the proposed sensor is more sensitive to shear forces than normal forces.

## 1. Introduction

Automated force measurement is a critical requirement for intelligent automation systems. Many one-dimensional (1D) force sensors based on micro-electromechanical systems (MEMS) techniques have been developed in recent years, including strain gauge-based force sensors, piezoresistive force sensors, capacitive force sensors, piezomagnetic force sensors, optical force sensors, and electroactive force sensors [1]. Furthermore, various researchers have demonstrated multi-dimensional force/torque sensors using multi-component structures [2,3]. Vázsonyi et al. [4] presented a three-dimensional (3D) sensor for detecting normal and shear forces incorporating a rectangular rod and an etched membrane. Sieber et al. [5] proposed a triaxial force sensing device consisting of a MEMS sensor attached to a three degree-of-freedom (3DOF) nanomanipulator. Cappelleri et al. [6] developed a vision-based sensor for micro-robotic systems consisting of a CCD camera and an elastic mechanism with a known force-deflection response. Kim [7] constructed a 3D force sensor for intelligent grippers based on five parallel plate-beams.

In general, the methods described above are designed for micro-force detection. However, in many engineering fields, e.g., shipbuilding, aerospace, and aviation, the ability to measure heavy 3D forces and loads remains a significant challenge. This problem is generally addressed using some form of parallel sensing mechanism since such structures have the advantages of stability, a high loading capability, zero error accumulation, and a high accuracy [8,9,10,11,12,13,14,15,16]. Dwarakanath and Venkatesh [17] proposed a simple “joint-less” parallel mechanism for force-torque sensing, in which the degrees of freedom of the structure were constrained by linearly-independent line constraints. Ranganath et al. [18] designed a force/torque sensor based on a Stewart platform with a near-singular configuration and optimized the structure in such a way as to achieve a well-conditioned transformation between the input and output forces. Liu and Tzo [19] investigated the measurement isotropy and sensitivity of a six-component force sensor consisting of four identical T-shaped bars. Yao et al. [20] conducted a theoretical and experimental investigation into the isotropy performance of a pre-stressed force sensor based on a Stewart platform. In later studies, the same group proposed a spatially isotropic configuration for the sensor [21] and a task-oriented method for its design [22]. However, in force/torque sensors based on a Stewart platform, the sensing mechanisms attached to the limbs are easily damaged under heavy loads. Thus, Liang and Wang [23] developed a humanoid robot ankle and integrated a force/torque sensor based on an orientation parallel kinematic mechanism. Lu et al. [24] designed and analyzed a novel force/torque sensor for a hybrid hand with three fingers.

In many of the methods described above, the effective measuring capability, or resolution, is deliberately sacrificed to a certain extent in order to increase the number of measurable force/torque components. However, some applications require more sensitivity to shear forces than normal forces and high-resolution measurement of only the exerted force (i.e., not the torque). For example, those aimed at measuring the 3D ground reaction force (GRF) in a human’s gait, since shear forces of GRF will be the critical effect that makes the human move forward, fall laterally, or be unbalanced during walking or running motions [25]. For another example, capturing the exerted shear force with precision is even more important than the normal (axial) force in measuring with the force sensor of articulated arm coordinate measuring machines (AACMMs) [26] or coordinate-measuring arms (CMAs) [27]. Accordingly, the present study proposes a novel 3D force sensor which measures only the 3D force. The sensor is based on a parallel mechanism. Thus, the transformation between the exerted 3D force and the mechanism posture can be established from a position analysis and the principles of static equilibrium. However, in a parallel mechanism, a particular equilibrium position of the end effecter is not necessarily associated with a unique solution of the posture. Accordingly, in the proposed sensor, the parallel structure comprises three pairs of parallel links such that each equilibrium position of the end effecter is constrained to a unique posture solution. Moreover, the torque effect acting on the end effecter is eliminated through the use of ball-socket joints in the parallel links. Finally, a constraint is added to the position analysis to ensure that the solution is continuously transformed from the initial posture of the sensor. Consequently, the magnitude of the applied 3D force can be determined by measuring the associated rotational angles of the three parallel links using rotary encoders.

The remainder of this paper is organized as follows: Section 2 describes the basic structure of the proposed 3D force sensor; Section 3 derives the transformation function and sensitivity index of the sensor; Section 4 presents and discusses the simulation results for the measurement capability and sensitivity of the sensor; and, finally, Section 5 presents some brief concluding remarks.

## 2. Mechanism of 3D Force Sensor

**Figure 1:**
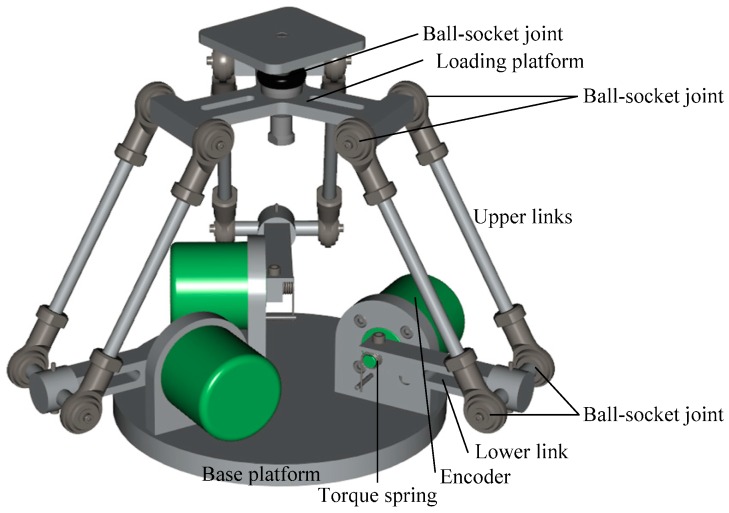
Structure of proposed 3D force sensor.

Figure 1 illustrates the novel 3D force sensor proposed in this study. As shown, the sensor consists mainly of a base platform, three linkages, and a loading platform. Each linkage comprises a pair of upper links and a single lower link, where the upper and lower links are connected by ball-socket joints. The two upper links are parallel to one another and are connected to the loading platform by a further set of ball-socket joints. One rotational degree of freedom exists between the lower link and the base platform, i.e., the torque spring and encoder are actuated by the rotational motion of the lower link. The torque spring is implemented using a leaf circular spring with a high and constant spring stiffness coefficient.

**Figure 2:**
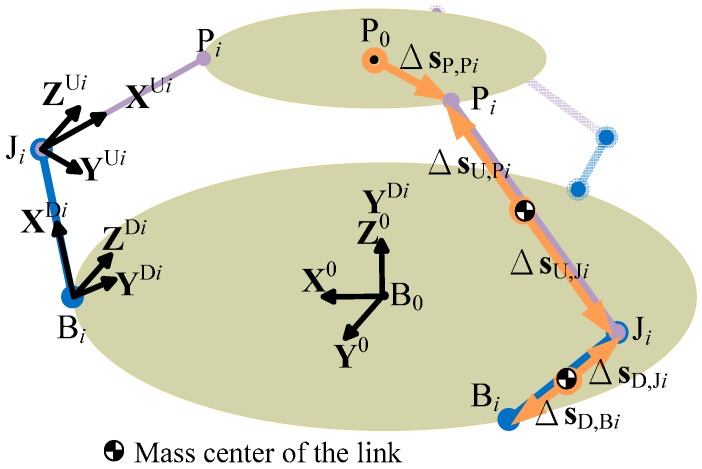
Definitions of points and vectors in proposed 3D force sensor.

Let B_0_ and P_0_ be the center points of the base and loading platforms, respectively; and B*_i_*, J*_i_*, and P*_i_* be the connection points between the platforms and links, where the suffix *i* denotes the *i*-th linkage, *i* = 1, 2, 3 (see Figure 2). In analyzing the position/posture of the sensor structure, the angular position of rotational joint B*_i_* is denoted as *θ_i_*, while the two rotational angles of the ball-socket joint J*_i_* are denoted as *α_i_* and *β_i_*, respectively. Due to the configuration of the sensor, the rotational axes of *α_i_* and *θ_i_* are parallel to one another and orthogonal to that of *β_i_*. As a result, the loading platform remains parallel to the base platform at all times and can move with three translational degrees of freedom.

## 3. Transformation Function and Sensitivity Index

Based on the definition of points B_0_, P_0_, B*_i_*, J*_i_*, and P*_i_* for the *i*-th linkage, *i* = 1, 2, 3 (see Figure 2), let the following parameters be introduced: (1) *γ_i_*: the angular position from X_0_ to vector B_0_B*_i_*, where *γ_i_* is equal to 0°, 120°, and −120° for *i* = 1, 2, 3, respectively, due to the triangular symmetrical arrangement of the three linkages; (2) *r*_B_ and *r*_P_: the lengths of vectors B_0_B*_i_* and P_0_P*_i_*, respectively; and (3) *L*_D_ and *L*_U_: the lengths of the lower and upper links, respectively. Based on the geometric relations between the links, and the constraints imposed by the parallel configuration of the sensor structure, the position of P_0_ can be determined mathematically as:

(1) $${P_{0}}^{i}=\left[\begin{array}{c} \cos\gamma_{i}\left(r_{B}+L_{D}\cos\theta_{i}+L_{U}\cos\left(\theta_{i}+\alpha_{i}\right)\cos\beta_{i}-r_{P}\right)-L_{U}\sin\gamma_{i}\sin\beta_{i}\\ \sin\gamma_{i}\left(r_{B}+L_{D}\cos\theta_{i}+L_{U}\cos\left(\theta_{i}+\alpha_{i}\right)\cos\beta_{i}-r_{P}\right)+L_{U}\cos\gamma_{i}\sin\beta_{i}\\ L_{D}\sin\theta_{i}+L_{U}\sin\left(\theta_{i}+\alpha_{i}\right)\cos\beta_{i} \end{array}\right]$$

Due to the parallel configuration, the same solution is obtained for each position vector **P**_0_*^i^*, *i* = 1, 2, 3, i.e., **P**_0_^1^ = **P**_0_^2^ = **P**_0_^3^ = [*P*_x_ *P*_y_ *P*_z_]^T^. Thus, the nine variables (*θ_i_*, *α_i_* and *β_i_* for *i* = 1, 2, 3) can be solved as:

(2) $$\theta_{i}=\cos^{-1}(\frac{{Μ}_{i}}{\sqrt{{{Κ}_{i}}^{2}+{\rho_{i}}^{2}}})+\lambda_{i}$$

(3) $$\beta_{i}=\sin^{-1}\left(\frac{-P_{x}\sin\gamma_{i}+P_{y}\cos\gamma_{i}}{L_{U}}\right)$$

(4) $$\alpha_{i}=\cos^{-1}\left(\frac{{\left(P_{x}\cos\gamma_{i}+P_{y}\sin\gamma_{i}-r_{B}+r_{P}\right)}^{2}+{\left(-P_{x}\sin\gamma_{i}+P_{y}\cos\gamma_{i}\right)}^{2}+{P_{z}}^{2}-{L_{D}}^{2}-{L_{U}}^{2}}{2L_{D}L_{U}\cos\beta_{i}}\right)$$

where: $\frac{{Κ}_{i}}{\sqrt{{{Κ}_{i}}^{2}+{\rho_{i}}^{2}}}=\cos\lambda_{i}$, ${Κ}_{i}=-2\left(r_{B}-r_{P}\right)L_{D}+2P_{x}\cos\gamma_{i}L_{D}+2P_{y}\sin\gamma_{i}L_{D}$, $\rho_{i}=2P_{z}L_{D}$, ${Μ}_{i}=-2P_{x}\cos\gamma_{i}\left(r_{B}-r_{P}\right)-2P_{y}\sin\gamma_{i}\left(r_{B}-r_{P}\right)+P_{x}{}^{2}+P_{y}{}^{2}+P_{z}{}^{2}+L_{D}{}^{2}-L_{U}{}^{2}+{\left(r_{B}-r_{P}\right)}^{2}$.

To establish the relation between the input force and the sensor posture, let the following vectors be introduced for each linkage: Δ**s**_P,P*i*_, Δ**s**_U,P*i*_, Δ**s**_U,J*i*_, Δ**s**_D,J*i*_, and Δ**s**_D,B*i*_, where each vector represents the vector from the mass center of the link to the connection point (see Figure 2), and can be formulated in terms of the link parameters and the posture variables, *θ_i_*, *α_i_*, and *β_i_*. Assuming that the mass centers are located at the mid-point positions of the links, the five vectors can be derived as:

(5) $$\Delta s_{P,Pi}=r_{P}\left[\begin{array}{c} \cos\gamma_{i}\\ \sin\gamma_{i}\\ 0 \end{array}\right]$$

(6) $$\Delta s_{U,Pi}=-\Delta s_{U,Ji}=\frac{L_{U}}{2}\left[\begin{array}{c} \cos\gamma_{i}\cos\left(\theta_{i}+\alpha_{i}\right)\cos\beta_{i}-\sin\gamma_{i}\sin\beta_{i}\\ \sin\gamma_{i}\cos\left(\theta_{i}+\alpha_{i}\right)\cos\beta_{i}+\cos\gamma_{i}\sin\beta_{i}\\ \sin\left(\theta_{i}+\alpha_{i}\right)\cos\beta_{i} \end{array}\right]$$

(7) $$\Delta s_{D,Ji}=-\Delta s_{D,Bi}=\frac{L_{D}}{2}\left[\begin{array}{c} \cos\gamma_{i}\cos\theta_{i}\\ \sin\gamma_{i}\cos\theta_{i}\\ \sin\theta_{i} \end{array}\right]$$

In accordance with the principles of static equilibrium, the force acting on the loading platform can be derived as:

(8) $$f_{x}+\sum_{i=1}^{3}f_{Pi,x}=0,\;f_{y}+\sum_{i=1}^{3}f_{Pi,y}=0,\;f_{z}+\sum_{i=1}^{3}f_{Pi,z}+m_{P}g_{0}=0$$

(9) $$\sum_{i=1}^{3}\left(r_{P}\sin\gamma_{i}f_{Pi,z}\right)=0,\;\sum_{i=1}^{3}\left(-r_{P}\cos\gamma_{i}f_{Pi,z}\right)=0,\;\sum_{i=1}^{3}\left(-r_{P}\left(\cos\gamma_{i}f_{Pi,x}+\sin\gamma_{i}f_{Pi,y}\right)\right)=0$$

where *m*_P_ is the mass of the loading platform; **g** is the gravity vector, i.e., **g** = [0 0 *g*_0_]^T^ m/s^2^; **f**_load_ is the 3 × 1 force vector applied to the loading platform along the X-, Y-, and Z-directions, respectively, i.e., **f**_load_ = [*f_x_* *f_y_* *f_z_*]^T^; and **f**_P*i*_ is the force acting at connection point P*_i_* and is denoted as **f**_P*i*_ = [*f*_P*i,x*_ *f*_P*i,y*_ *f*_P*i,z*_]^T^. The torque acting at point B*_i_*, which causes rotational motion of the torque spring and encoder, can be derived as:

(10) $$n_{Bi}=\left(\Delta s_{U,Pi}-\Delta s_{U,Ji}+\Delta s_{D,Ji}-\Delta s_{D,Bi}\right)\times f_{Pi}-\left(-\Delta s_{U,Ji}+\Delta s_{D,Ji}-\Delta s_{D,Bi}\right)\times m_{Ui}g+\Delta s_{D,Bi}\times m_{Di}g$$

where *m*_P_, *m*_U*i*_, and *m*_D*i*_ are the masses of the loading platform, upper links, and lower link of the *i*-th linkage, respectively. The torque along the rotational axis of *θ_i_* is given by:

(11) $${\left(n_{Bi}\right)}^{T}\cdot Z^{Di}=K_{T}\cdot \Delta \theta_{i}$$

where **Z**^D*i*^ is the rotational axis of *θ_i_*, *K*_T_ is the stiffness coefficient of the torque spring, and Δ*θ_i_* is the rotational angle measured by the encoder. The rotational angles of the three encoders can be used to estimate the 3D loading force by solving Equations (8)–(11), and can be expressed as the following nonlinear function of the exerted force:

(12) $$\Delta \theta_{i}=G_{i}\left(f_{x},f_{y},f_{z}\right)\;\mathrm{for}\;1\leq i\leq 3$$

In general, the sensitivity of a transducer is evaluated as the rate of variation of the output relative to the input. However, this traditional definition cannot be applied to the force sensor proposed in the present study since the transformation function of the proposed sensor has a nonlinear form and the system has multiple (rather than single) inputs and outputs (i.e., the system is a multi-input-multi-output (MIMO) system). Accordingly, the sensitivity of the proposed sensor is described instead using a Jacobian matrix $\hat{J}$, in which the net-force dependent problem is resolved by dividing the exerted forces by the respective Euclidean norms. In other words, the components of $\hat{J}$ appearing in the *i*-th row are the derivatives of the nonlinear function *G_i_* with respect to the exerted force, **f**_load_, i.e.,

(13) $${\hat{J}}_{i}=\frac{\partial G_{i}}{\partial f_{\mathrm{load}}}\;\mathrm{for}\;1\leq i\leq 3$$

The measurement sensitivities of the sensor for exerted forces in the X-, Y-, and Z-directions (referred to as the X-, Y-, and Z-sensitivities, respectively, and denoted as *ν_x_*, *ν_y_*, and *ν_z_*) are defined as the Euclidean norms of each column in $\hat{J}$, i.e.,

(14) $$\hat{J}=\left[\begin{array}{ccc} {\hat{J}}_{x} & {\hat{J}}_{y} & {\hat{J}}_{z} \end{array}\right]$$

(15) $$\nu_{y}=\left\|{\hat{J}}_{y}\right\|$$

Meanwhile, the variation of the sensor sensitivity for forces exerted in different directions is defined as the sensitivity diversity index (*v_d_*) and is derived in accordance with the spectral norm of the Jacobian matrix $\hat{J}$ and its inverse ${\hat{J}}^{-1}$ as follows:

(16) $$\nu_{d}={\left\|\hat{J}\right\|}_{2}-{\left({\left\|{\hat{J}}^{-1}\right\|}_{2}\right)}^{-1}=\sqrt{\lambda_{\max}}-\sqrt{\lambda_{\min}}$$

where *λ*_max_ and *λ*_min_ are the largest and smallest eigenvalues of $\left({\hat{J}}^{*}\cdot \hat{J}\right)$, respectively, and ${\hat{J}}^{*}$ is the conjugate transpose of $\hat{J}$.

## 4. Simulation Results and Discussion

The formulations presented in the previous section for the transfer function and sensitivity of the proposed 3D force sensor were implemented in a MATLAB (MathWorks, Inc., Natick, MA, USA) program. Simulations were then performed to investigate the force measuring capability of the proposed device and its measurement sensitivity with respect to the forces exerted in the X-, Y-, and Z-directions.

### 4.1. Effective Measuring Capability

From the previous investigation [28], the parallel mechanism will have the maximum workspace while the lengths of the upper links are twice as long as the lower links. Thus, in evaluating the measuring capability of the sensor, the link lengths were set as *r*_B_ = *r*_P_ = 45 mm, *L*_D_ = 60 mm and *L*_U_ = 120 mm, and these links are assumed as rigid bodies. In addition, the stiffness coefficient of the torque spring was set as *K*_spring_ = 0.7 Nm/deg, while the initial angular position of the rotational joint B*_i_* in the absence of an applied torque was set as *θ_i_* = 45°. Finally, the working range of the rotational angle, Δ*θ_i_*, was assumed to be 90°, i.e., from −55° to 35°.

**Figure 3:**
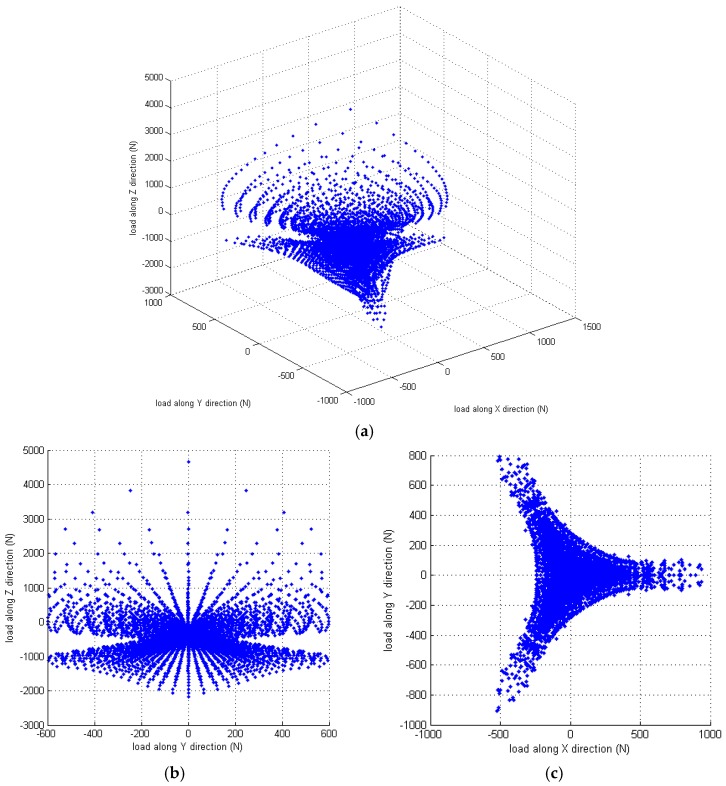
Effective measuring capability of the 3D force sensor: (**a**) overall view of the effective measuring distribution; (**b**) measuring distribution in cross-section *f*_x_ = 0 N; and (**c**) measuring distribution in cross-section *f*_z_ = 0 N.

Figure 3 shows the corresponding results obtained for the effective measuring capability of the sensor in the X-, Y-, and Z-directions. It is seen that the distribution of the measurable 3D force has a triangular symmetrical characteristic, which mimics that of the sensor mechanism configuration.

### 4.2. Sensitivity and Sensitivity Diversity Index

**Figure 4:**
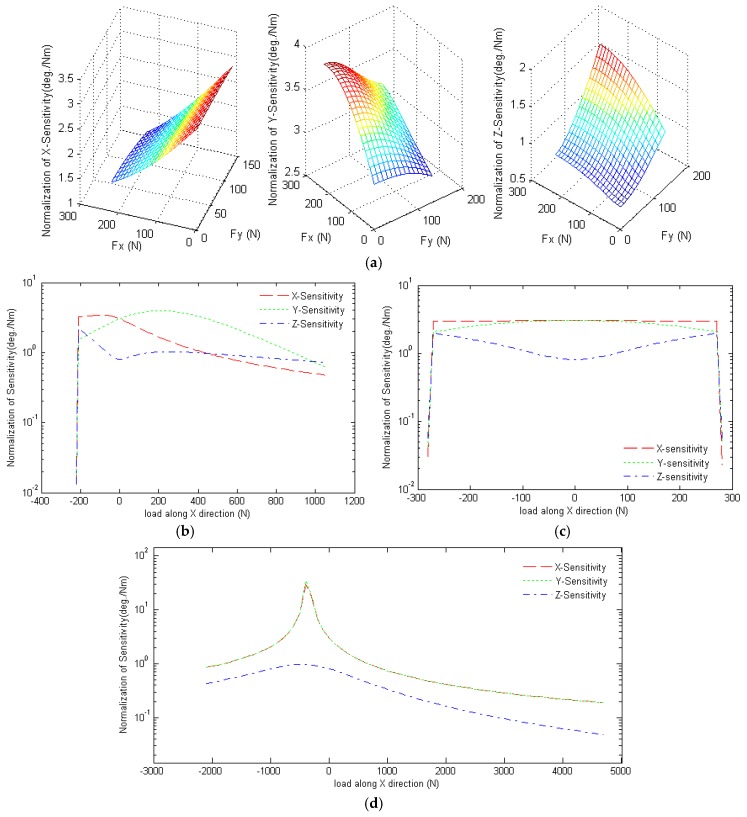
Normalization of X-, Y-, and Z-sensitivities of the proposed sensor under: (**a**) horizontal force; (**b**) horizontal force along the X-direction only; (**c**) horizontal force along the Y-direction only; and (**d**) vertical force.

Since the sensitivities are directionally proportional to the lever arm, in accordance with the deriving formulation of sensitivities in Equations (10)–(15), we normalize the sensitivities by dividing the length of the lower link to decouple the size effect. Figure 4 shows the normalization of the X-, Y- and Z-sensitivities of the proposed sensor for various input load conditions. As described in Section 3, the inputs and outputs of the sensor are related via a nonlinear transfer function and, hence, the sensitivity depends on the static equilibrium posture. Based on the structure of the 3D force sensor, the equivalent lever arm of the horizontal force (i.e., the force acting along the X- or Y-direction) is approximately equal to the sum of the vertical components of vectors $\vec{B_{i}J_{i}}$ and $\vec{J_{i}P_{i}}$. Similarly, the equivalent lever arm of the vertical force (i.e., the force acting along the Z-direction) is approximately equal to the difference in the horizontal components of the vector $\vec{B_{i}J_{i}}$ and $\vec{J_{i}P_{i}}$. For most static equilibrium postures of the sensor, the equivalent lever arm of the horizontal exerted force is larger than that of the vertical exerted force. Moreover, the higher sensitivity is caused by the higher effective driving torque, which varies in proportion to the equivalent lever arm. Therefore, the device has a greater measuring sensitivity for horizontal forces than for vertical forces, other than in the central triangular region, i.e., *f*_x_ > 450 N (see Figure 4). In other words, the sensor is more sensitive to shear forces than normal forces over most of the measurable force region. In this regard, the sensor differs from traditional force sensors, which generally exhibit greater a sensitivity toward forces acting in the normal direction.

The greater sensitivity of the proposed sensor to shear forces is due to the greater efficacy of horizontal forces in exciting the rotational angle of the encoders. When the force sensor is subject to a vertical force only, the postures of the three linkages are the same as those under static equilibrium conditions. Given such a symmetrical posture, the transmission angles of the horizontal force are greater than those of the vertical force. Therefore, the X- and Y-sensitivities of the sensor are greater than the Z-sensitivity (see Figure 4d).

**Figure 5:**
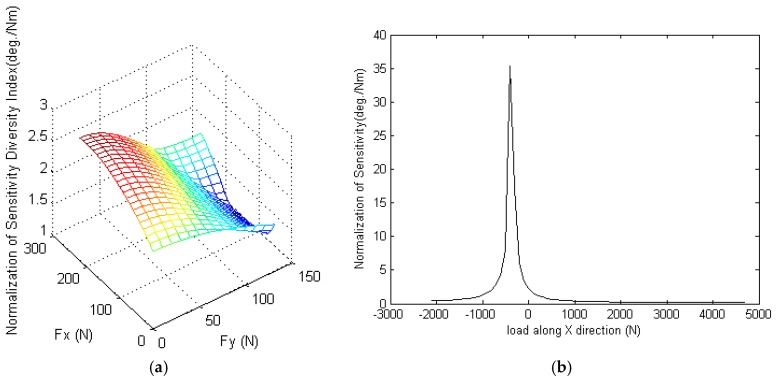
Normalization of the sensitivity diversity index under: (**a**) horizontal force; and (**b**) vertical force.

Figure 5 shows the simulation results for the normalization of the sensitivity diversity index *ν*_d_ of the sensor. It is seen that normalization of *ν*_d_ has a value of less than 2.943 deg/Nm given the application of a horizontal force. However, the normalization of the sensitivity diversity index has a value of up to 35.3 deg/Nm under a vertical force due to the near-orthogonal transmission angles of the applied force along the X- and Y-directions.

### 4.3. Design Variable Analysis

**Figure 6:**
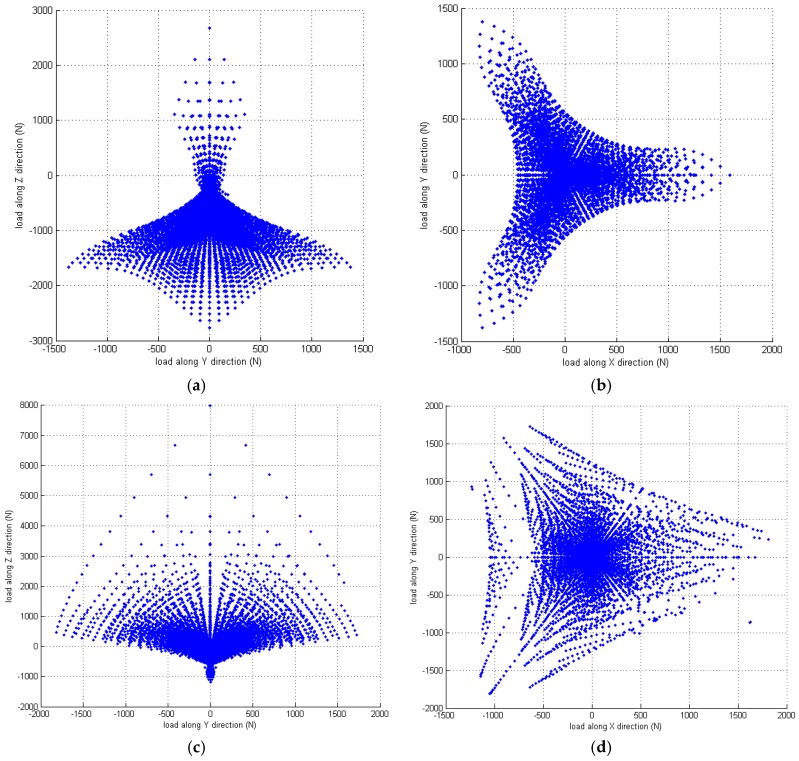
Effective measuring capability of proposed sensor given the different initial angular positions of torque spring: (**a**) measuring distribution in cross-section *f*_x_ = 0 N with the initial angular of 60°; (**b**) measuring distribution in cross-section *f*_z_ = 0 N with the initial angular of 60°; (**c**) measuring distribution in cross-section *f*_x_ = 0 N with the initial angular of 20°; (**d**) measuring distribution in cross-section *f*_z_ = 0 N with the initial angular of 20°.

In order to discuss the design variables, we define the same parameters as the above example, i.e., *r*_B_ = *r*_P_ = 45 mm, *L*_D_ = 60 mm, *L*_U_ = 120 mm, *K*_spring_ = 0.7 Nm/deg, and links are rigid bodies. However, the initial angular position and stiffness coefficient of the torque spring are separately changed to find their effect. For the effect of the angular position of the torque spring, it is assumed that the rotational angle of the links is limited to the range of −10° to 90° (i.e., −10 deg ≤ *θ_i_* ≤ 90 deg) in designing the 3D force sensor. In addition, the rotational angles of the encoders, Δ*θ_i_*, are assumed to be positive under the effects of a tensile exerted force (i.e., *f*_z_ > 0 N), and negative under a compressive exerted force (i.e., *f*_z_ < 0 N). Figure 6 shows the effective 3D force measuring capability of the proposed sensor given initial angular positions of the torque spring equal to 60° and 20°, respectively. From the simulation results, we can find that the effective measuring capability of the sensor for a tensile force increases as the initial angular position of the torque spring decreases, while that for a compressive force varies proportionally with the initial angular position of the torque spring. In addition, there is an asymmetrical plot existing at the point of *f*_x_ = 1628 N and *f*_y_ = −863.2 N. This may be caused by a numerical analysis error since the point is close to the workspace boundary of the parallel mechanism. The solution of the nonlinear equations would be solved with a different configuration of the force sensor. Thus, it may appear that the measurement error is close to the measurable boundary. Furthermore, we define different stiffness coefficients of the torque spring to find its effect, and Table 1 shows the X-, Y-, and Z-sensitivities of the proposed sensor under horizontal and vertical forces, respectively, given different values of the torque spring stiffness coefficient. From the results, the sensitivities are inversely proportional to the stiffness coefficient, while the direction of the exerted forces are the same with the sensitivity, i.e., X- and Y-sensitivities for horizontal forces, and Z-sensitivity for vertical forces. This is because the larger stiffness would decrease the resolution of the transformation between the force and the angular position.

## 5. Conclusions

A novel force sensor has been proposed for measuring 3D forces only (i.e., not the torque). The sensor is based on a parallel structure. Analytical expressions have been derived for the transformation function and sensitivity index of the proposed device based on a position analysis and the principles of static equilibrium. The simulation results have shown that the sensor has a greater effective measuring capability than traditional force sensors. In addition, the sensitivity of the proposed device for exerted horizontal forces is greater than that for exerted vertical forces. In other words, the sensor is more sensitive to shear forces than normal forces and, thus, differs from traditional force sensors, for which the reverse is generally true. Notably, the effective measuring capability of the device for tensile or compressive forces can be tuned by modifying the initial angular position of the torque springs used to activate the encoders. In addition, the sensitivity of the device can be adjusted by using torque springs with a different stiffness coefficient.

The previous studies about force sensors are applied to the structural design to decouple the horizontal and vertical forces, and the forces and torques can be divided into component forces measured by the inserted force sensor. Nevertheless, in the proposed force sensor, we estimate the exerted force by measuring three angular positions caused by three torques which are equal to the exerted force multiplied by the lever arm. In addition, the equivalent lever arm of the horizontal force is approximately equal to the sum of the vertical components of two links, and the equivalent lever arm of the vertical force is approximately equal to the difference in the horizontal components of two links. The lever arm of the horizontal force is larger than that of the vertical force. Thus, the horizontal (shear) force will be more sensitive than the vertical (normal) force.

Furthermore, the critical factors which caused the different performances between simulations and the physical prototype are assumptions of rigid links and clearanceless joints. Nevertheless, the effect of the flexible links and joint clearance can be estimated via finite element analysis (FEM) [29], and the design of the non-spherical ball joint or the optimization links can be adopted to improve the loading performance [30]. In addition, it is rare for the spring to have a constant stiffness coefficient in all extension/compression ranges, and the relationship of the stiffness coefficient and extension/compression length needs to be distinguished. In further research, these effects will be considered in simulations to increase the accuracy compared with the actual force sensor prototype. In practice, it is very difficult to isolate the effects of the individual installation variables. However, according to the sensitivity analysis presented in [31], the positioning accuracy of the end effector in parallel manipulators is particularly sensitive to the link length, and the effect of the assembly defect (e.g., lack of parallelism, alignments, etc.) is less sensitive. Thus, the tolerance requirement of link dimension for manufacturing the actual prototype needs to be less than the maximum tolerance Δ*l_i_*/*l_i_* = 10^−3^.

## Figures and Tables

**Table 1:** Sensitivity of the proposed sensor given different stiffness coefficient values of the torque spring.

Stiffness Coefficient	Horizontal Force			Vertical Force		
	ν_x, max_	ν_y, max_	ν_z, max_	ν_x, max_	ν_y, max_	ν_z, max_
0.3 (Nm/deg)	0.426	0.427	0.274	1.559	1.614	0.136
0.7 (Nm/deg)	0.181	0.182	0.120	1.809	2.132	0.058
1.5 (Nm/deg)	0.080	0.084	0.057	2.501	3.720	0.027
